# Development of a Novel Methotrexate-Loaded Nanoemulsion for Rheumatoid Arthritis Treatment with Site-Specific Targeting Subcutaneous Delivery

**DOI:** 10.3390/nano12081299

**Published:** 2022-04-11

**Authors:** Parvathy Suresh, Mounir M. Salem-Bekhit, Hafsa Palathum Veedu, Sultan Alshehri, Sreeja Chandrasekhar Nair, Sarah I. Bukhari, Vidya Viswanad, Ehab I. Taha, Ram Kumar Sahu, Mohammed M. Ghoneim, Ibrahim Elbagory

**Affiliations:** 1 Department of Pharmaceutics, Amrita School of Pharmacy, Amrita Vishwa Vidyapeetham, AIMS Health Science Campus, Kochi 682041, India; sparvathy09@yahoo.in (P.S.); hafsapv1@gmail.com (H.P.V.); sreeju2u@gmail.com (S.C.N.); 2 Kayyali Chair for Pharmaceutical Industry, Department of Pharmaceutics, College of Pharmacy, King Saud University, P.O. Box 2457, Riyadh 11451, Saudi Arabia; mbekhet@ksu.edu.sa; 3 Department of Microbiology and Immunology, Faculty of Pharmacy, Al-Azhar University, Cairo 11884, Egypt; 4 Department of Pharmaceutics, College of Pharmacy, King Saud University, Riyadh 11451, Saudi Arabia; sbukhari@ksu.edu.sa (S.I.B.); eelbadawi@ksu.edu.sa (E.I.T.); 5 Department of Pharmaceutical Science, Assam University (A Central University), Silchar 788011, India; 6 Department of Pharmacy Practice, College of Pharmacy, AlMaarefa University, Ad Diriyah 13713, Saudi Arabia; mghoneim@mcst.edu.sa; 7 College of Pharmacy, Northern Border University, Arar 1321, Saudi Arabia; ibrahim.elbagory@nbu.edu.sa

**Keywords:** rheumatoid arthritis, methotrexate, anti-arthritic activity, nanoemulsion, hemocompatibility, MTT assay, stability studies

## Abstract

Rheumatoid arthritis (RA) is a systemic, chronic autoimmune disease that causes disability due to progressive inflammation and destruction of the tissues around the joints. Methotrexate is mainly used to prevent the progression of joint destruction and reduce the deformity. The major challenge in treating RA with methotrexate is the systemic side effects that limit dose escalation. Hence, a novel formulation of a methotrexate-loaded nanoemulsion for subcutaneous administration was developed that aims to deliver methotrexate into the system via the lymph. The methotrexate-loaded nanoemulsion was prepared by using the aqueous-titration method. The prepared nanoemulsion was investigated for particle size, surface charge, surface morphology, entrapment efficiency, DSC (differential scanning colorimetry), drug release, hemocompatibility assay, and cytotoxicity, as well as anti-arthritic and stability studies. The vesicle size, zeta potential, PDI (polydispersity index), and entrapment efficiency of the optimized nanoemulsion were 87.89 ± 2.86 nm, 35.9 ± 0.73 mV, 0.27, and 87 ± 0.25%, respectively. The DSC study showed that the crystalline methotrexate was converted to an amorphous form and the drug was fully incorporated into the vesicles. After 72 h, the optimized nanoemulsion showed a drug release of 96.77 ± 0.63%, indicating a sustained-release dosage form. Cytocompatibility testing by MTT (3-(4,5-dimethylthiazol-2-yl)-2,5-diphenyl-2H-tetrazolium bromide) assay on macrophage cell lines showed that the nanoemulsion was non-toxic. The formulation showed significant anti-arthritic activity compared to the marketed drug solution. In addition, the nanoemulsion containing methotrexate remained stable for three months when stored at a low temperature. Since the nanoemulsion containing methotrexate has excellent physicochemical properties and lowers systemic side effects by targeted delivery, it is a desirable technology for subcutaneous drug delivery.

## 1. Introduction

Rheumatoid arthritis (RA) is a systemic autoimmune disease characterized by chronic inflammation of the joints and associated tissues. Although the inflammation is primarily associated with the joints, other organs in the body are also affected [1,2]. RA has a prevalence of approximately 0.24%, with a higher prevalence in females than males [3]. There is no clear cause for the disease, but genetic factors, environmental factors, and lifestyle changes trigger the inflammatory response in the body. Hormonal imbalance alters cortisol and androgen levels triggered by stress and leads to inflammatory response [4,5]. It is believed that the influx of inflammatory cells (T cells and B cells) into the pannus and synovial fluid is the cause of the pathology of RA leading to complete tissue destruction [6]. Inflammation causes an increase in the production of cytokines, which results in joint damage, synovitis, and edema. Synovial fluid contains a large number of immune cells, most of which are monocytes and mast cells. In addition, there are a small number of adaptive immune cells, such as plasma cells, Th1 (T-helper type 1), Th17, and B cells, which are involved in the inflammatory process. Further, the joint fluid contains high concentrations of antibodies against citrullinated proteins, which stimulates the release of complement proteins, which in turn trigger the onset of the inflammatory response [7].

Treatment aims to reduce joint inflammation and pain as well as tissue abnormalities and deformities in order to restore life’s quality. Corticosteroids, NSAIDs (non-steroidal anti-inflammatory drugs), and opioids are considered to be first-line drug therapies for inflammatory pain and swelling. These medications are given for a limited period and are intended to relieve the pain and swelling associated with inflammation. The prevention of further joint damage and reduction of deformity are the primary goals of therapy [8,9]. The nonbiologic drugs methotrexate, sulfasalazine, hydroxychloroquine, and leflunomide are the most commonly used disease-modifying antirheumatic drugs (DMARDs) [10]. Fewer people take gold salts, azathioprine, and cyclosporine, among other rare medications. In RA, biologic DMARDs consist of monoclonal antibodies and recombinant receptors that inhibit the production of cytokines that contribute to the prevention of inflammation [11,12].

**Figure 1:**
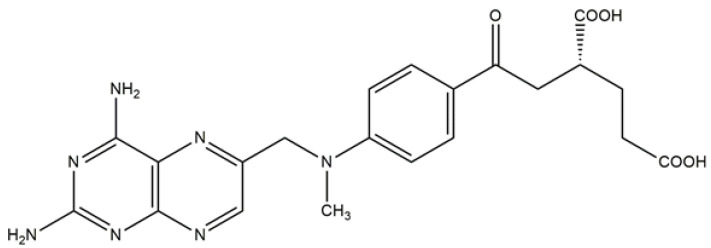
Chemical structure of methotrexate.

Methotrexate is the most important component of DMARDs, and its structure is shown in Figure 1. It is a folate derivative that suppresses pyrimidine and purine production [12]. Methotrexate reduces inflammation in RA by reducing the proliferation of inflammatory cells and lymphocytes, resulting in a decrease in the number of active inflammatory cells. Methotrexate increases the level of intracellular adenosine, which interacts with certain cell-surface receptors to inhibit the synthesis of the chemokines and leukotriene B4 as well as the regulation of synovial collagenase genes [13].

The systemic side effects of methotrexate, which limit dose escalation, are a significant obstacle in treating RA with this drug. The use of encapsulated carriers for targeted delivery may reduce the need for excessive and frequent dosing. When it comes to the pathophysiology of RA, the lymphatic system plays an important role [14,15]. Anti-inflammatory drugs can be delivered through the lymphatic system to improve bioavailability and therapeutic efficacy while reducing adverse effects [16]. Drugs can be more effectively targeted to the lymphatic system when encapsulated in carriers ranging in size from micro- to nanoscale. The use of encapsulated carriers, particularly nanoparticles, can contribute to delayed release while reducing the amount of drugs used [17,18].

When nanoparticle carriers are administered intravenously, they are consumed by reticular endothelial cells, preventing the drug from reaching the lymphatic system. Subcutaneous administration can be used for lymphatic targeting because of the permeability of capillaries, which allows the transport of smaller molecules [19]. Subcutaneous delivery of drug-loaded nano-molecules and their uptake into the lymph make it a suitable route that has a positive impact on the ongoing treatment profile of RA. Because of the above statement, it was planned to prepare and characterize a novel methotrexate-loaded nanoemulsion for the treatment of RA by targeted subcutaneous administration with improved bioavailability and therapeutic efficacy. The use of the nanoemulsion as a carrier can improve the bioavailability of methotrexate. The toxicity of the nanoemulsion as a carrier was studied using the cytotoxic and hemolysis assays to ensure that it was safe to use. In addition, CFA (complete Freund adjuvant)-induced arthritis in rats was used as a disease paradigm to evaluate the antirheumatic effect of methotrexate-loaded nanoemulsion.

## 2. Results and Discussions

### 2.1. Particle Size Study

**Figure 2:**
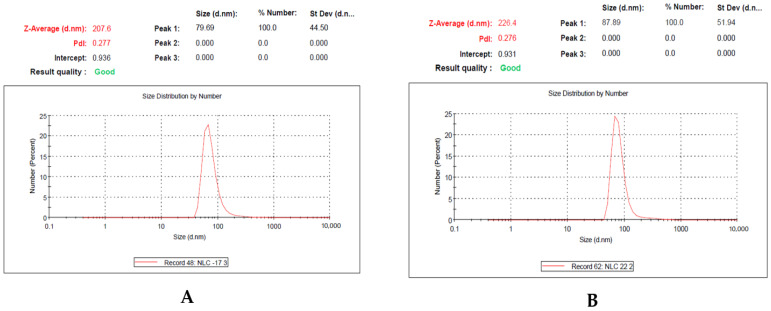
Data of particle-size distribution and zeta potential of the prepared nanoemulsion; (**A**): Nanoemulsion without drug; (**B**): Methotrexate-loaded nanoemulsion.

The size of the particles plays a significant influence in their uptake and retention in lymphoid tissue. When the particle size of different formulations was determined using a zetasizer by differential light scattering, a decrease in particle size was observed with the addition of a mixture of surfactant and co-surfactant. The optimized nanoemulsion loaded with methotrexate had a mean particle size of 87.89 ± 2.86 nm, whereas the particle size of the nanoemulsion without the drug was 76.39 ± 1.53 nm (Figure 2). The larger size of the nanoemulsion loaded with methotrexate compared to the nanoemulsion without the drug indicates that the drug was successfully loaded into the nanoemulsion. It has been documented that a nanoemulsion with a particle size smaller than 100 nm increases the transport of the drug through the lymphatic capillaries, which absorb the drug from the interstitial space [20]. Moreover, this particular size of the nanoemulsion can be easily absorbed and retained in the lymphoid tissue, resulting in improved therapeutic efficacy compared to conventional drugs. The PDI of the optimized nanoemulsion loaded with methotrexate was 0.27 (Figure 2). The nanoemulsion has smaller vesicles with a low PDI. The PDI reflects the potential of the size distribution of the vesicles in the nanoemulsion. In addition, the PDI is one of the most effective techniques with which to evaluate the homogeneity and stability of the nanoemulsion vesicles [21,22]. A higher PDI value of about 0.7 shows that the formulation has a very wide particle-size distribution, while values of 0.2 and below are mostly agreeable formulations. A PDI value of 0.3 and below is considered acceptable and indicates a uniform distribution of lipiodol vesicles [22]. The size distribution of the optimized nanoemulsion was in agreement with the particle size.

**Figure 3:**
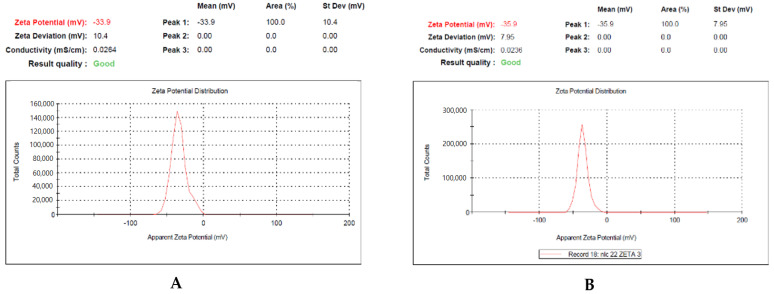
Data of zeta potential. (**A**): Nanoemulsion without drug; (**B**): Methotrexate-loaded nanoemulsion.

Zeta potential is an indicator of the stability of nanoemulsion. Determination of the zeta potential gave a value of −35.9 ± 0.73 mV for the optimized nanoemulsion loaded with methotrexate, while the zeta potential for nanoemulsion without drug was −33.9 ± 1.03 mV (Figure 3). The values of the zeta potential confirmed that the prepared nanoemulsion was negatively charged droplets with the capacity of higher lymphatic uptake and longer retention time. It measures the electrical repulsion force between the particles. It has been documented a higher zeta potential (>−30 mV) is advantageous to vesicle’s physical stability as it avoids accumulation between vesicles owing to electrostatic repulsion resulting in a stable emulsion. The low zeta potential of emulsions leads to coagulation or flocculation of vesicles, resulting in poor physical stability [23,24,25]. According to these findings, we concluded that the selected composition of drug, oil, and surfactant makes the nanoemulsion electrically stable.

### 2.2. Morphology of Particle 

**Figure 4:**
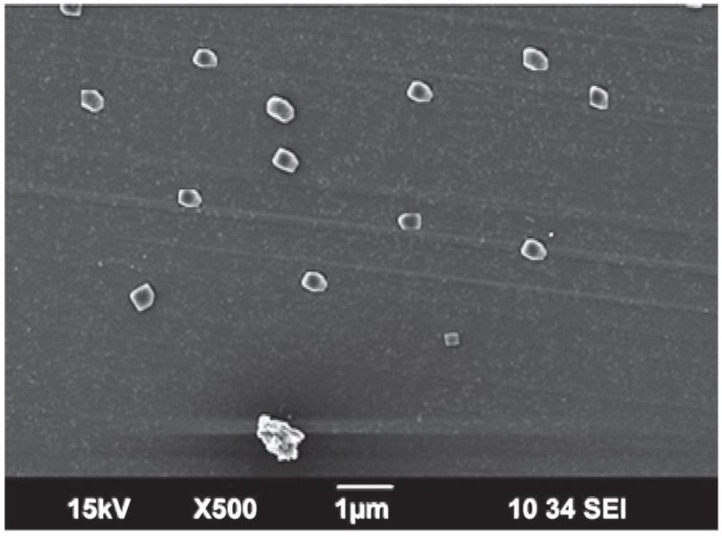
Representation of SEM findings of methotrexate-loaded nanoemulsion.

The SEM (scanning electron microscope) photograph of the methotrexate-loaded nanoemulsion shows that the particles have almost spherical morphology, are uniform and monodispersed (Figure 4). A nanoemulsion is a kinetically stable, dispersed system with particles of small droplet size. The spherical shapes indicate the stability of the system without aggregation and gravitational separation. This stability can be attributed to the presence of surface-active agents. The study conducted by Zhou et al. shows that the spherical nanoemulsion can rapidly penetrate the cell [26]. The results from SEM are consistent with the result of particle-size characterization. 

**Figure 5:**
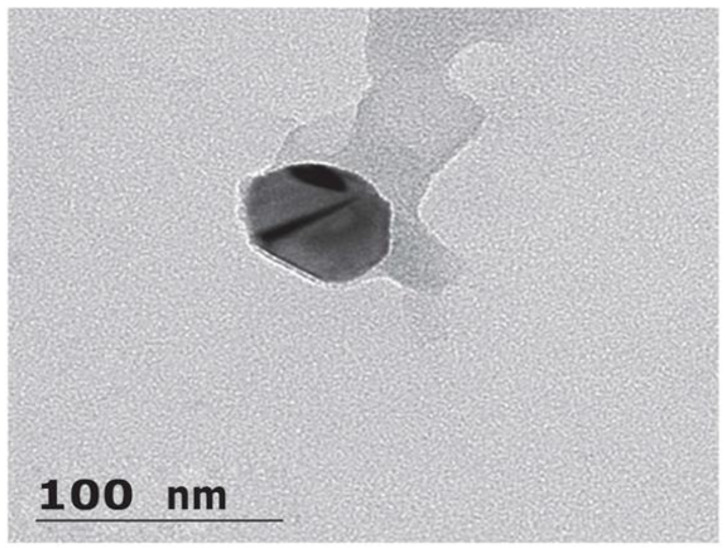
Representation of TEM findings of methotrexate-loaded nanoemulsion.

The TEM (transmission electron microscopy) image of methotrexate-loaded nanoemulsion shows a particle size of less than 100 nm with a spherical shape (Figure 5). The results of the particle size and SEM study support the findings of TEM. El-Refai et al. and Antil et al. documented a spherical and uniform distribution of vesicles in the nanoemulsion prepared with sesame oil and Tween 80 in their TEM study [27,28]. The results of TEM are consistent with the study in which the nanoemulsion was prepared with sesame oil and Tween 80. 

### 2.3. DSC Study 

**Figure 6:**
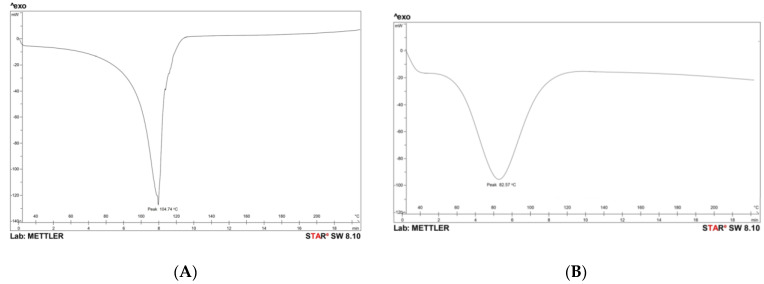
Differential scanning colorimetry of pure-drug and methotrexate-loaded formulations: (**A**) Pure drug; (**B**) Methotrexate-loaded formulation.

The DSC thermogram of methotrexate showed an endothermic peak at 104.74 °C, which is related to the melting point of the methotrexate, and a peak endothermic peak at 82.57 °C, which resembles the melting point of the methotrexate-loaded nanoemulsion (Figure 6). The DSC thermogram shows a sharp endothermic peak of methotrexate, proving its crystalline state. The endothermic peak of methotrexate did not appear in the DSC thermogram of the nanoemulsion, and the broadened thermal peak of the nanoemulsion confirms that methotrexate was fully incorporated into the vesicles. The different thermal peak of methotrexate and the nanoemulsion justifies that the drug is in an amorphous state. Moreover, as the temperature increases, there is an interaction between methotrexate and oil, which forms a complex that has a lower melting point (82.57 °C) than pure methotrexate [29,30].

### 2.4. Entrapment Efficiency of Nanoemulsion 

To determine the quantity of drugs successfully incorporated into the nanoparticles, the entrapment efficiency must be measured. The entrapment efficiency of the nanoemulsion was calculated using an indirect method by measuring the free drug available in the aqueous phase. The entrapment efficiency of the methotrexate-loaded nanoemulsion was found to be 87.25%, indicating high efficacy. The higher inclusion of methotrexate may be due to its stronger incorporation into the hydrophobic core. Antil et al. reported a 78% encapsulation efficiency of the metaxalone-loaded nanoemulsion containing sesame oil and Tween 80 [28]. The encapsulation-efficiency results are in agreement with the results of Antil et al.

### 2.5. In Vitro-Release Study 

**Figure 7:**
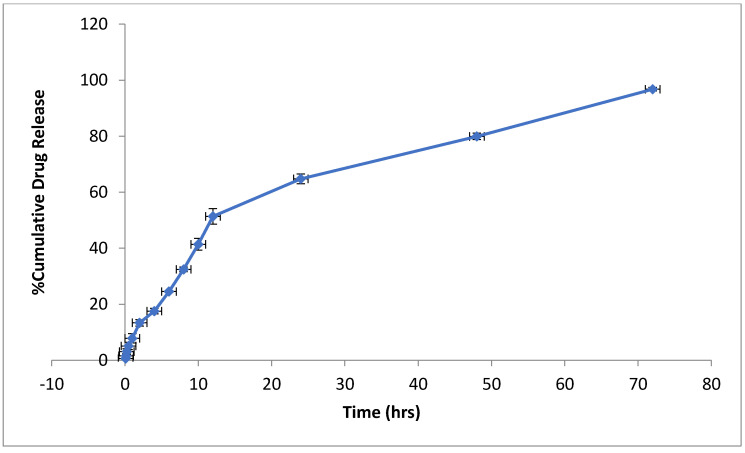
Graphical representation of drug release from the nanoemulsion loaded with methotrexate.

In vitro release from the methotrexate-loaded nanoemulsion was performed using a dialysis membrane that showed a biphasic release pattern. In this formulation, the drug was released in a burst at the beginning of the test, followed by a sustained release. The methotrexate-loaded nanoemulsion exhibited a 96.77% release after 72 h (Figure 7). The rapid release of the drug of 13.39 ± 1.14 from the nanoemulsion was observed during the first 2 h of the experiment. The suppressed drug release was observed after 2 h, indicating prolonged drug release from the nanoemulsion. The results are in agreement with other studies that addressed the biphasic release of methotrexate from the nanoemulsion. In this regard, Rashid et al. and Rathee et al. showed drug release of 72% after 20 h and 55% after 24 h, respectively [31,32,33]. Initially, the rapid release of the drug from the nanoemulsion could be caused by the methotrexate that is adsorbed on the droplet surface or dispersed in the surfactant. The sustained release indicates that the nano-sized drug is retained within the nanoemulsion droplet. In addition, the drug release in the nanoemulsion is also controlled by the interactions of the drug with the surfactants and its distribution between the aqueous and oil phases. The delayed drug release is of great interest for systemic delivery, especially for arthritis. The results suggest that the sustained release of the methotrexate-loaded nanoemulsion is a better formulation for the treatment of arthritis.

**Figure 8:**
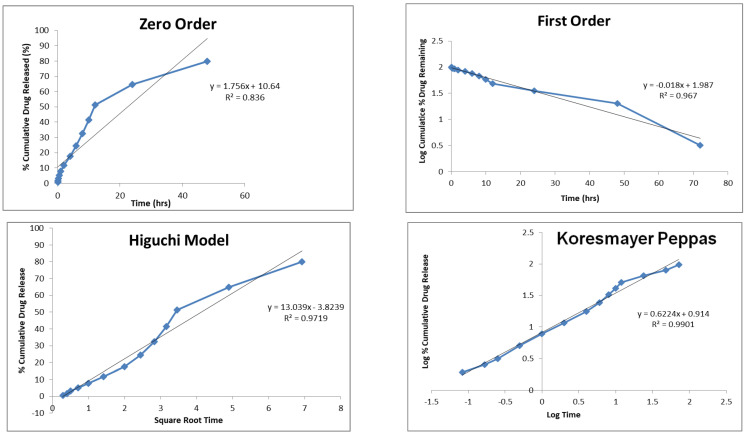
Graphical representation of kinetic-data analysis of methotrexate-loaded nanoemulsion.

The order and mechanism of drug release from the nanoemulsion were investigated by applying the drug-release data to the zero-order, first-order, Higuchi and Korsmeyer–Peppas models. It was found that the drug release from the nanoemulsion was most closely associated with the Higuchi model, based on the R^2^ values. The diffusion of methotrexate from the oily core and interface is retarded by the aqueous medium, which acts as a physical barrier to the release of the drug due to its poor solubility in water, a likely cause of prolonged release. It would appear that drug release is controlled by diffusion if the *n* value from the Korsmeyer–Peppas equation is used (Figure 8).

### 2.6. Hemocompatibility Analysis of Nanoemulsion 

**Figure 9:**
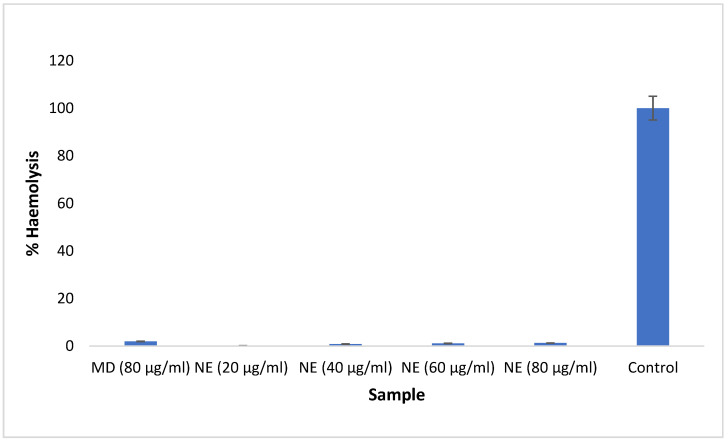
Percentage hemolysis of methotrexate-loaded nanoemulsion and pure methotrexate solution. NE: Methotrexate-loaded nanoemulsion; MD: Marketed drug.

The nanoemulsions loaded with methotrexate were tested and showed negligible hemolysis after a prescribed incubation period. The different concentrations of methotrexate-loaded nanoemulsion showed hemolysis ranging from 0.14 to 1.27% (Figure 9). The percentage of hemolysis in the samples does not increase significantly as the concentration of nanoemulsion increases. The marketed methotrexate drug solution exhibited 1.98% hemolysis. It has been documented that materials with hemolysis greater than 5% are considered hemolytic, while materials with hemolysis between 5 and 2% are termed as slightly hemolytic, and materials with less than 2% hemolysis are considered very hemocompatible [34]. The results indicate that the nanoemulsion is less than 2% hemolytic. This suggests that the prepared nanoemulsion is very hemocompatible and has no toxic effect on blood vessels. The nanoemulsion loaded with methotrexate showed less hemolysis compared to the marketed drug solution, indicating that the nanoemulsion is much safer compared to the conventional formulation.

### 2.7. Cytotoxic Assay

**Figure 10:**
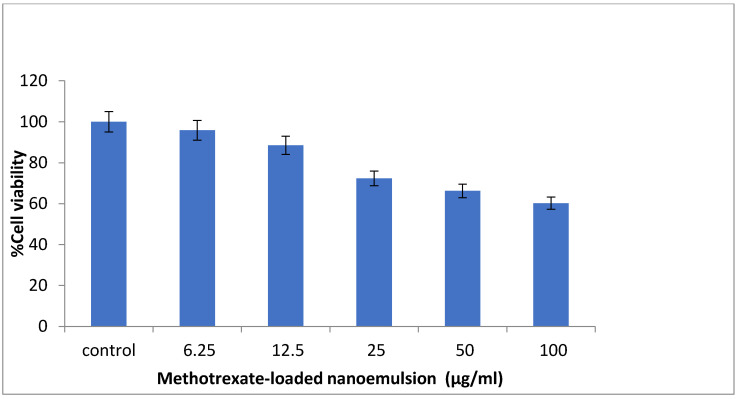
Graphical representation of in vitro cytotoxic effect of methotrexate-loaded nanoemulsion.

The MTT study was used to detect the living cell’s metabolic activity in order to evaluate the cell viability of methotrexate-loaded nanoemulsion against the macrophage cell line RAW 264.7. The cell viability of RAW 264.7 cells at various concentrations of methotrexate-loaded nanoemulsion ranged from 95.79% to 60.21% (Figure 10). It was found that the cell viability of RAW 264.7 cells decreased in direct proportion to the concentration of methotrexate-loaded nanoemulsion. This indicates that the higher concentration of nanoemulsion induces mild cell cytotoxicity. The RAW 264.7 cell line exhibited a cell viability of less than 88% at concentrations of 6.25 and 12.5 μg/mL in the nanoemulsion. The results of hemolysis support the results of the cytotoxicity assay. In addition, the findings indicate that the higher dose of methotrexate-loaded nanoemulsion may cause mild toxicity to cells, while the lower dose is safe.

### 2.8. In Vivo Anti-Arthritic Activity of Nanoemulsion 

**Figure 11:**
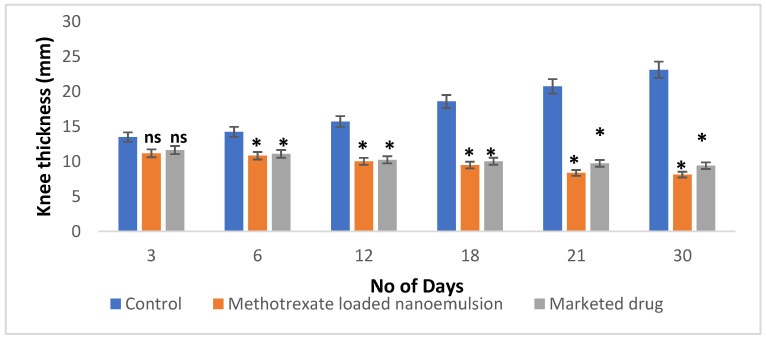
Knee-edema studies in complete-Freund-adjuvant-induced arthritis model. Results are shown as mean ± SEM (*n* = 6) and analyzed by one-way ANOVA followed by Tukey’s test; ns: nonsignificant; * *p* < 0.05 significance difference to compared to control group.

The methotrexate-loaded nanoemulsion was intended to investigate the anti-arthritic efficacy by measuring the potential of the nanoemulsion to inhibit CFA-induced knee edema in rats. It showed a significant reduction in inflammation in the methotrexate-loaded nanoemulsion and pure methotrexate groups over 30 days compared to the control group. Figure 11 demonstrated a continuing increase in knee inflammation by the disease control animals. The animal treated with the methotrexate-loaded nanoemulsion (10 mg/mL per kg body weight) and marketed methotrexate significantly (*p* < 0.05) decreased the knee inflammation compared to control animals on days 6–30. The highest knee edema was noted during the 30 days of study in control animals (23.11 ± 0.14 mm), whereas the edema was controlled by the methotrexate-loaded-nanoemulsion-treated (8.12 ± 0.13 mm) and marketed-drug-treated (9.40 ± 0.39 mm) animals. The methotrexate-loaded nanoemulsion showed a greater reduction in swelling compared to the marketed drug, suggesting that the methotrexate-loaded nanoemulsion has improved anti-arthritic potential compared to the conventional dosage form.

**Figure 12:**
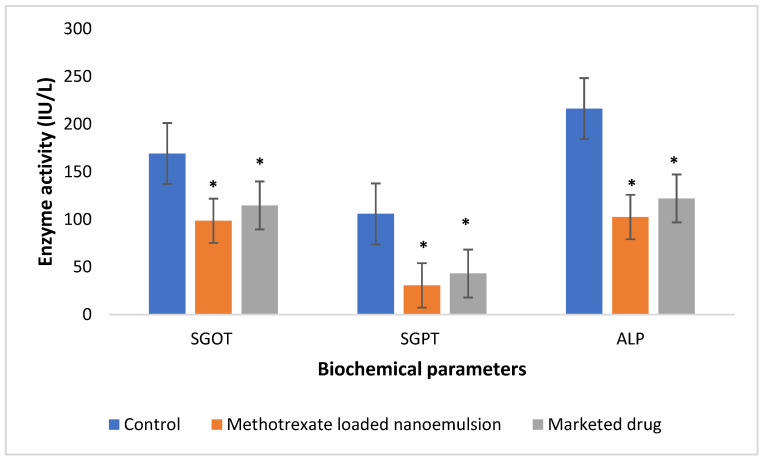
Liver-function tests for methotrexate-loaded nanoemulsion and pure methotrexate solution. Results are shown as mean ± SEM (*n* = 6) and analyzed by one-way ANOVA followed by Tukey’s test; * *p* < 0.05 significance difference compared to control group.

The liver-protective properties of the methotrexate-loaded nanoemulsion were studied after the end of the experiment. When methotrexate is administered for a prolonged period or at higher doses, it causes liver toxicity, but in the present study, the formulations were administered to the rats only for a short time, so the possibility of liver toxicity was low. The function of hepatic marker enzymes SGOT (serum glutamic oxaloacetic transaminase), SGPT (serum glutamic pyruvic transaminase) and ALP (alkaline phosphatase) were used to evaluate the cellular architecture of the animals with CFA-induced arthritis. SGOT, SGPT, and ALP were elevated in the control group of animals. Hepatic marker enzymes were significantly (*p* < 0.05) decreased in methotrexate-loaded-nanoemulsion- and marketed-drug-treated animals (Figure 12). It was observed that the methotrexate-loaded nanoemulsion exhibited greater liver-protective properties than the marketed drug solution. The results indicated that the CFA-induced animals experienced a significant effect on hepatic enzyme activity, while the methotrexate-loaded nanoemulsion and marketed methotrexate significantly decreased hepatic enzyme activity compared with the control group. The results suggest that the nanoemulsion loaded with methotrexate has better liver-protective properties compared to the marketed drug, as the function of liver marker enzymes such as SGOT, SGPT and ALP is much closer to the healthy liver function. This confirms that the methotrexate-loaded nanoemulsion enables targeted drug delivery with minimal access of the drug to the liver and also prevents the metabolism of the drug by the liver cell, resulting in improved therapeutic efficacy. The in vivo anti-arthritic study confirms the capability of the methotrexate-loaded nanoemulsion to lessen systemic toxicity as well as improve anti-arthritic efficacy compared to the usual dosage forms.

**Figure 13:**
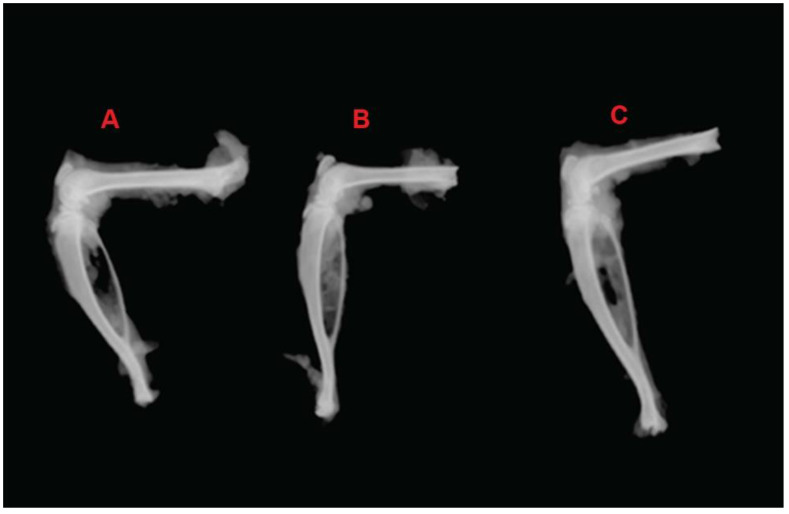
Radiographic analysis of the knees with the administration of the formulation. (**A**): FCA-induced arthritis, control group; (**B**): Methotrexate-loaded nanoemulsion; (**C**): marketed drug solution.

A radiographic analysis of the animals on day 30 showed bone devastation and soft-tissue swelling along with joint-space narrowing in the CFA-induced control group, indicating subchondral erosion in the arthritic state. The methotrexate-loaded nanoemulsion and marketed drug solution showed no bone destruction and normal soft-tissue swelling in the animals (Figure 13). When RA occurs, radiography is an important diagnostic technique that can be used to determine the extent of arthritis. The early stages of arthritis are characterized by soft-tissue swelling and inflammation, whereas the later stages are characterized by subchondral erosions and narrowing of the joint space. The methotrexate-loaded nanoemulsion and marketed drug solution showed a significant reduction in joint destruction and soft-tissue damage in the animals. The potent anti-arthritis effect of the methotrexate-loaded nanoemulsion was subsequently validated and confirmed by the radiographic study of the knee joints of the animals.

### 2.9. Stability Studies 

**Figure 14:**
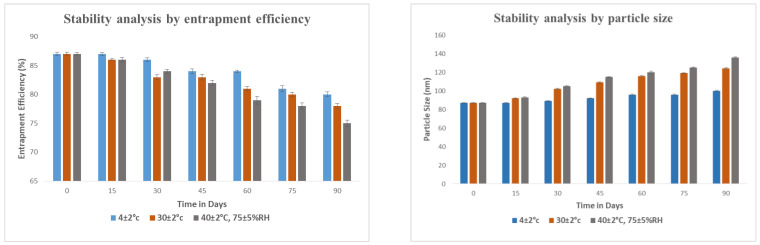
Graphical representation of stability study of methotrexate-loaded nanoemulsion according to particle size and entrapment efficiency.

To verify the stability properties of the methotrexate-loaded nanoemulsion, the stability studies of the nanoemulsion were carried out at refrigerated, room and elevated temperatures for three months. The particle size and entrapment efficiency changed significantly during storage at room temperature and elevated temperature. The possible mechanism for this was the polymorphic transformation of methotrexate. No changes in particle size and entrapment efficiency were observed at cooling temperatures (Figure 14). This indicates that the methotrexate-loaded nanoemulsion formulation was more stable under cooling temperature. Therefore, the methotrexate-loaded nanoemulsion is an excellent formulation for curing RA as it mitigates systemic side effects and improves treatment efficacy compared to the conventional dosage form.

## 3. Materials and Methods

### 3.1. Materials

The sample of the methotrexate injection was acquired from the leading IP Pharmacy center, AIMS, Kochi, Kerala, India. The surfactant, namely sesame oil, was purchased from the local grocery. Nice Chemicals, located in Ernakulam, Kerala, India, provided the Tween 80 for this study. The DMEM (Dulbecco’s modified Eagle’s medium) was procured from the well-known supplier thermo fisher scientific India Pvt Ltd., Ernakulum, Kerala, India. 

### 3.2. Methodology

#### 3.2.1. Formulation of Methotrexate-Loaded Nanoemulsion

Aqueous-phase titration was used to prepare the methotrexate-loaded nanoemulsion. In this procedure, the distilled water was gently added to the oil mixture and mixed dropwise with dynamic stirring until the resulting formulation appeared transparent and clear. Methotrexate solution at the concentration of 50 mg/mL was prepared and 0.2 mL solution was mixed into 3 mL of sesame oil, and then a mixture of 7 mL of Tween 80 and 2 mL of ethanol was slowly added while stirring. The resultant solution was vortexed for about 5 min using a vortex mixer. The aqueous phase, which consisted of distilled water, was added dropwise while constantly stirring at 500 rpm until a nanoemulsion was formed [35].

#### 3.2.2. Particle Characteristics 

The droplet size, polydispersity index (PDI), and zeta potential of the prepared nanoemulsion were determined using the Malvern Zetasizer (Nano ZS Malvern Instruments Ltd., Mavern, UK). After appropriate dilution, the nanoemulsions were sonicated and the droplet size was measured. The goal of this study was to assess the stability of the prepared nanoemulsion by measuring the droplet size, distribution, and zeta potential [35,36].

#### 3.2.3. Particle Surface Morphology

The morphology and size were also determined by scanning electron microscopy (SEM) (VEGA 3, TESCAN, Brno-city, Kohoutovice, Czech Republic) and transmission electron microscopy (TEM) (TALOS, Thermo fisher scientific India Private Limited, Mumbai, India), respectively. The methotrexate-loaded nanoemulsion was diluted 10-fold with distilled water and dried at room temperature on 200 mesh film grids. For SEM analysis, samples were fixed in the sample holder with double-sided adhesive tape, and pictures were taken at a voltage of 5 kV. For TEM, samples were stained with a 2% phosphotungstic-acid solution and then dried for 2 min before being viewed under an electron microscope at 100 kV [37,38].

#### 3.2.4. Differential Scanning Colorimetry (DSC) 

The DSC (DSC 204F1 Phoenix, NETZSCH-Gerätebau GmbH, Selb, Germany) method was used to determine the change in physical properties and temperature of the optimized nanoemulsion. The sample was placed in an aluminum pan sealed with perforated lids and heated in the temperature range of 40–300 °C at a constant rate of 10 °C per minute. In addition, the inert atmosphere was created by a nitrogen purge of 50 mL/min. It assesses the stability of the formulation [39].

#### 3.2.5. Entrapment Efficiency 

In a slight modification of the method reported by Sarheed et al., a cellulose dialysis membrane technique was used to estimate the encapsulation of methotrexate in the nanoemulsion by assessing the free drug available in the aqueous phase. Before the experiment, the membrane was soaked overnight in phosphate-buffer solution (pH 7.4). By capping both ends, the nanoemulsion with 10 mg of drug incorporated was kept in the dialysis membrane. Phosphate-buffer solution and ethanol were mixed in a ratio of 80:20, and 100 mL was added into the receptor compartment. The dialysis membrane was placed in this 100 mL receptor compartment and shaken with a mechanical stirrer for 24 h. Aliquots were withdrawn from the receptor compartment and the amount of free drug crossing the dialysis membrane was measured by UV spectroscopy (UV-1700, Shimadzu, Kyoto, Japan) at 303.5 nm [40]. The following equation was used to measure the entrapment efficiency:

$$\mathrm{Entrapment}\;\mathrm{Efficiency}=\frac{\mathrm{Quantification}\;\mathrm{sample}\;\mathrm{drug}-\mathrm{Quantification}\;\mathrm{of}\;\mathrm{free}\;\mathrm{drug}\;\mathrm{in}\;\mathrm{the}\;\mathrm{solution}}{\mathrm{Quantification}\;\mathrm{sample}\;\mathrm{drug}}\times 100$$

#### 3.2.6. In Vitro Drug Release and Kinetic-Modeling Study 

The cellulose dialysis membrane technique was used for in vitro drug release from the methotrexate-loaded nanoemulsion. The membrane was well cleaned and soaked overnight in phosphate buffer (pH 7.4) before use. Then, 10 mg of the drug was added to the membrane and both ends were tightly sealed. The dialysis membrane was kept in a beaker containing a phosphate-buffer solution with a pH of 7.4 and a temperature of 37 ± 1 °C throughout the study. The solution in the beaker was continuously stirred with a magnetic stirrer at 50 rpm. Samples were withdrawn from the beaker at specific intervals and the volume was maintained by adding the same amount of medium. The withdrawn aliquots were analyzed spectrophotometrically at 303.5 nm. Various kinetic models were developed to measure the release order and mechanism of release based on these results [41].

#### 3.2.7. Hemocompatibility Analysis 

The hemocompatibility study of the methotrexate-loaded nanoemulsion was performed to determine the suitability of the composition and the effect on red blood cells. To perform the test, a slight modification of the method reported by Roka and colleagues was employed. A volume of 5 mL of blood was drawn from healthy volunteers and an anticoagulant was added to prevent blood clotting. The blood sample was further diluted with a phosphate buffer with a pH of 7.4. The different concentrations of methotrexate-loaded nanoemulsion (20, 40, 60, and 80 µg/mL) and marketed drug (80 µg/mL) were added separately to the diluted blood samples and incubated for 24 h. The sample was then centrifuged at 3500 rpm for 10 min at 4 °C. The supernatant containing blood cells was placed in a microtiter plate, and absorbance was measured at 540 nm using an Elisa plate reader [42]. The percentage of hemolysis was calculated by using the following formula:

$$\%\mathrm{Hemolysis}=\frac{\mathrm{Blood}\;\mathrm{cells}\;\mathrm{in}\;\mathrm{the}\;\mathrm{supernatant}\;\mathrm{of}\;\mathrm{the}\;\mathrm{sample}\;\mathrm{solutions}}{\mathrm{Blood}\;\mathrm{cells}\;\mathrm{in}\;\mathrm{the}\;\mathrm{distilled}\;\mathrm{water}}\times 100$$

#### 3.2.8. Cytotoxic Assay 

MTT assay on RAW 264.7 (macrophage) cell lines was used for the evaluation of the in vitro cytotoxicity effect of the methotrexate-loaded nanoemulsion. The RAW 264.7 cell lines were cultured in DMEM (Dulbecco’s modified Eagle’s medium) media which was supplemented with 10% FBS, L-glutamine, sodium bicarbonate, and antibiotics at 37 °C maintaining humidification of 5% CO_2_. The different concentrations of 6.25 µg/L, 12.5 µg/L, 25 µg/L, 50 µg/L, and 100 µg/L of nanoemulsion were prepared by diluting with 5% DMEM. These samples were added to the cell culture and incubated at 37 °C in a humidity of 5% CO_2_. The normal saline solution was considered as the control solution for the measurement of cell viability. 

The samples were incubated for 24 h, after which 30µL of reconstituted MTT solution was added to all test and control wells. The plate was shaken slowly and then incubated at 37 °C for 4 h to maintain the humidification of 5% CO_2_ in the incubator. After completion of incubation, the supernatant was taken out, and added to 100µL of MTT solubilization solution. To solubilize the formazan crystals, the wells were carefully agitated by pipetting up and down. A microplate reader was used to assess the absorbance of samples at a wavelength of 570 nm, which signifies the optical density (OD) [43]. 

The percentage of viable cells was measured by using the following equation:

$$\%\;\mathrm{of}\;\mathrm{viability}=\frac{\mathrm{Mean}\;\mathrm{OD}\;\mathrm{of samples}}{\mathrm{Mean OD of control}}\times 100$$

#### 3.2.9. In Vivo Anti-Arthritic Activity

Approval for the animal study was obtained from the Central Animal Facility of the Amrita Institute of Medical Sciences (IAEC/2017/3/9) for the animal experiments. Experiments were conducted on adult male Sprague Dawley rats weighing about 200–250 g in order to determine the anti-arthritic effect. The animals were segregated into three groups of six animals each and kept in polypropylene cages at a temperature of 23 ± 2 °C. Intra-articular injection of 1 mg/mL CFA was injected to all groups of animals to induce the Chronic Arthritis condition. After that, methotrexate-loaded nanoemulsion (10 mg/mL) and marketed methotrexate drug (10 mg/mL) were administered subcutaneously once a week for 30 days to groups II and III, respectively, while no treatment was given to group I. Using a digital micrometer, the knee circumference (in mm) of each group was determined every day for 30 days. After 30 days of testing, retro-orbital blood was drawn from the rats and the serum was separated to assess the functions of liver enzyme markers (SGPT, SGOT, and ALP). In addition, radiographs of the knee joints of rats were performed to evaluate the effect of marketed methotrexate and methotrexate-loaded nanoemulsion on the severity of arthritis in FCA-induced rats [44,45].

#### 3.2.10. Stability Studies 

For three months, methotrexate-loaded nanoemulsion was stored at three different temperatures: refrigerator temperature (4 ± 2 °C), room temperature (30 ± 2 °C), and accelerated temperature (40 ± 2 °C, 75 ± 5% RH). Particle size (PS) and entrapment efficiency (EE) were evaluated at 15-day intervals over 90 days to check the changes in physical and chemical stability of the nanoemulsion [41].

## 4. Conclusions

In the treatment of RA, methotrexate is a DMARD that is considered a first-line therapy. There are only a small number of drug-delivery systems on the market that provide significant pharmacotherapy for rheumatoid arthritis. Methotrexate formulations currently on the market have been associated with systemic side effects. Therefore, a methotrexate-loaded subcutaneous nanoemulsion that delivers the drug exclusively to the lymphatic system was developed and studied in vitro and in vivo in order to minimize adverse effects and improve therapeutic efficacy. The nanoemulsion was found to encapsulate a significant amount of methotrexate with a smaller particle size and shape suitable for subcutaneous injection. Physicochemical studies confirmed that the methotrexate-loaded nanoemulsion exhibited homogeneous nanosized droplets and a stable formulation. In vitro drug-release studies confirmed the sustained release of the drug from the nanoemulsion. The results of hemolysis and cytotoxicity studies showed that the nanoemulsion is safer for systemic circulation at lower concentrations. The anti-arthritic study of the nanoemulsion in CFA-induced animals showed improved anti-arthritic activity compared to the marketed drug. The preliminary studies of the methotrexate-loaded nanoemulsion suggest that the formulation may be able to improve the lymphatic transport of drugs after systemic administration. In addition, this formulation may reduce drug doses while minimizing adverse effects on cell integrity. It can be concluded that methotrexate-loaded nanoemulsion is a superior formulation for the treatment of RA as it mitigates systemic side effects and improves treatment efficacy compared to the conventional dosage form. In the future, the drug-uptake study in the lymph node will demonstrate the ability of the nanoemulsion to deliver drugs subcutaneously into the lymphatic circulation.

## Data Availability

Not applicable.
